# Synthesis, Characterization, and Gas Adsorption Performance of Amine-Functionalized Styrene-Based Porous Polymers

**DOI:** 10.3390/polym15010013

**Published:** 2022-12-20

**Authors:** Katerina Setnickova, Karel Jerabek, Tomas Strasak, Monika Mullerova, Vera Jandova, Karel Soukup, Roman Petrickovic, Hui-Hsin Tseng, Petr Uchytil

**Affiliations:** 1 Institute of Chemical Process Fundamentals, Czech Academy of Science, Rozvojova 135, 165 02 Prague, Czech Republic; 2 Department of Occupational Safety and Health, Chung Shan Medical University, Taichung 402, Taiwan; 3 Department of Environmental Engineering, National Chung Hsing University, Taichung 402, Taiwan

**Keywords:** CO_2_ adsorption, hyper-crosslinked porous polymer, amine impregnation, dendrimer, gas separation

## Abstract

In recent years, porous materials have been extensively studied by the scientific community owing to their excellent properties and potential use in many different areas, such as gas separation and adsorption. Hyper-crosslinked porous polymers (HCLPs) have gained attention because of their high surface area and porosity, low density, high chemical and thermal stability, and excellent adsorption capabilities in comparison to other porous materials. Herein, we report the synthesis, characterization, and gas (particularly CO_2_) adsorption performance of a series of novel styrene-based HCLPs. The materials were prepared in two steps. The first step involved radical copolymerization of divinylbenzene (DVB) and 4-vinylbenzyl chloride (VBC), a non-porous gel-type polymer, which was then modified by hyper-crosslinking, generating micropores with a high surface area of more than 700 m^2^ g^−1^. In the following step, the polymer was impregnated with various polyamines that reacted with residual alkyl chloride groups on the pore walls. This impregnation substantially improved the CO_2_/N_2_ and CO_2_/CH_4_ adsorption selectivity.

## 1. Introduction

Anthropogenic carbon dioxide is considered among the main cause of the greenhouse effect responsible for global warming. More than 65% of anthropogenic CO_2_ is generated from burning fossil fuels during the energy production. To limit the increase in the Earth’s temperature, reducing greenhouse gas emissions is necessary. Therefore, development of new technologies for producing clean energy with minimal CO_2_ emissions is necessary. CO_2_ can be reduced using carbon capture and storage (CCS) technologies based on the capture, transport, and storage of CO_2_ [1,2].

CCS technologies are considered potentially promising in reducing CO_2_ emissions, and thereby relieving climate change [3,4]. Adsorption is considered an effective alternative for CO_2_ capture, offering possible energy savings compared to other established separation technologies [5,6].

In recent years, the research interest in solid adsorbent materials has increased. Compared to traditional separation procedures, they are easy to operate and offer relatively low energy requirements [7].

Different types of solid adsorbents have been investigated for CO_2_ capture, such as carbon-based materials, zeolites, metal organic frameworks, and porous polymers [8,9,10,11].

Considering the direction of society towards a circular economy of materials, the use of plastic waste as a source of functional materials for various applications has become an important challenge for the future [12,13,14].

Examples of recovery of waste plastic include carbon-based materials created by carbonization [15], functional polymers prepared by post-synthetic chemical functionalization [16,17], and numerous other low-molecular products created after the decomposition of polymers [18,19].

In recent years, considerable attention has been paid to the preparation of hyper-crosslinked polymer adsorbents (HCLPs), a family of robust microporous organic materials with numerous benefits for gas storage, such as high surface area, permanent porosity, good thermal stability, and easy large-scale production [20,21,22,23,24].

Polystyrene-based polymers form a significant part of waste polymers because polystyrene is a cheap polymer precursor for the preparation of HCLPs [12,25]. Owing to the minimal biological degradation of polystyrene in nature [22], it causes many ecological problems; therefore, methods for its re-use should be investigated.

Styrenic materials, either monomers or polymers, can be easily functionalized via electrophilic aromatic substitution. Furthermore, an increased affinity of PS for CO_2_ compared to N_2_ or CH_4_ can be expected owing to the dipole interactions of phenyl groups containing π-electrons with quadrupolar CO_2_. Therefore, HCLPs based on PS and their amine group-modified forms have recently been investigated as efficient and low-cost CO_2_ adsorbents [12,26,27,28,29,30,31,32,33,34,35,36,37,38]. For example, Fu et al. [27,28] synthesized several types of HCLPs from waste expanded PS using different cross-linking agents; the best achieved CO_2_ absorption was 2.5 mmol g^−1^ and CO_2_/N_2_ separation efficiency of 38 at 273 K. The cross-linking of aromatic polymers was found to improve the textural properties of the prepared materials and enhance their CO_2_ adsorption capacity [28,33,34,35]. Wu et al. [36] prepared HCPs of different porosities from commercial PS; the porous structure of the HCLPs was tuned by varying the ratio of PS and cross-linker. The maximal CO_2_ adsorption capacity was 1.12 mmol g^−1^ at 298 K and a pressure of 1 atm.

The amine functionalization of PS adsorbents was studied by Lee et al. [32]. The influence of the length of the polyamines used in modifying the porous polymer adsorbents on their structure and selective CO_2_ adsorption was studied. After amination, the prepared materials showed increased CO_2_ adsorption capacities (the values were similar for all used polyamines, up to 1.86 mmol g^−1^ at 273 K and 1 bar) and higher CO_2_/N_2_ selectivity; however, the surface area of the modified polymers was considerably reduced. Similarly, Gaikar et al. [37] investigated the CO_2_ uptake and CO_2_/N_2_ selectivity of PS-based adsorbents by modifying chloromethylated PS with *N*-heterocyclic compounds.

Conversely, Merchán-Arenas et al. [38] found only a moderate CO_2_ uptake of 1.05 mmol g^−1^ at 273 K for amine-tethered PS adsorbents synthesized from waste expanded PS.

This study focuses on the synthesis of hyper-crosslinked vinylbenzyl chloride (VBC)-divinylbenzene (DVB) microporous material and its functionalization using the amino group-containing substances, simple amines, and larger dendrimer molecules to investigate their potential for application in CO_2_ capture and gas separation. The main objective of the work is the preparation of a series of hyper-crosslinked polymers, the characterization of their physicochemical properties and the investigation of the CO_2_ adsorption performance and selectivity of materials over other gases and to analyze the effect of modification by amino groups containing substances of different sizes.

## 2. Materials and Methods

### 2.1. Materials

All the chemicals required for synthesis of the hyper-crosslinked VBC-DVB microporous material and its functionalization were purchased from Sigma Aldrich, Prague, Czech Republic. Divinylbenzene (DVB; 80 vol% technical grade) and 4-vinylbenzyl chloride (VBC) were purified before use by passing them through a column of basic alumina. Other commercially available reagents were used as received.

Pure gases used for the adsorption experiments were purchased from Linde Gas with a stated purity of at least 99.995%.

### 2.2. Synthesis of Porous Polymer

**Figure 1:**
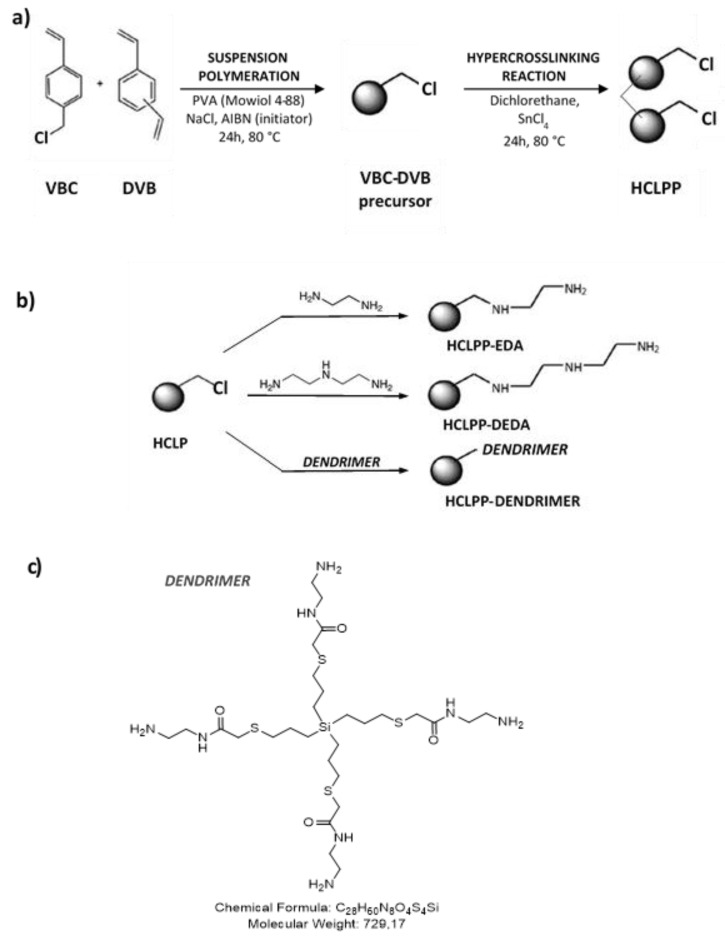
Scheme of (**a**) the synthesis of the hyper-crosslinked porous polymer (HCLPP) and (**b**) post-polyamine functionalization by ethylenediamine, diethylenetriamine, and dendrimer (**c**).

A hyper-crosslinked VBC-DVB microporous polymer was prepared and functionalized in two steps, as shown in the schema in Figure 1a. The first step involved the suspension polymerization of DVB and 4-vinylbenzyl chloride (VBC). The resulting polymer was then hyper-crosslinked under the conditions shown in Figure 1a. During this operation, certain chloromethyl groups within the polymer network were transformed into crosslinked methylene bridges. This additional crosslinking (hyper-crosslinking) induces phase separation within the polymer gel, resulting in the creation of pores between the hyper-crosslinked nodules. However, the conversion of a chloromethyl group into a methylene bridge can proceed only if a neighboring polymer chain is available in the vicinity of the chloromethyl group. The probability of finding such a reaction partner in the surface layer of the nodules, which is on the walls of the newly created pores, is low; hence, unreacted chloromethyl groups are available for further modification on the pore walls [24]. In the second step, various polyamine species were applied to react with the alkyl chloride groups and functionalize the pore surface to improve the selectivity of the material for CO_2_ (see Figure 1b).

#### 2.2.1. Preparation of Hyper-Crosslinked Porous Polymer Sample (HCLPP)

The polymerization was performed as follows: 20.2 g (130 mmol) of VBC was mixed with 1 g of technical grade (80%) DVB (corresponding to 5% crosslinking of the polymer matrix) and 0.6 g of AIBN (initiator) was added. An aqueous solution (1000 mL) containing 10 g of polyvinyl alcohol (Mowiol 4-88, MW = 31000, Sigma Aldrich, Prague, Czech Republic) and 50 g of sodium chloride was introduced into the reactor. The polymerization mixture was poured, the reactor was closed, and the mixture was stirred at 400 rpm. After one hour of stirring at room temperature, the temperature was raised to 80 °C, and the mixture was allowed to react for 24 h. Then, the reactor was opened, and the polymer was separated in a sieve, washed with ethanol, and dried. The resulting partially bonded balls were broken in a grinding mill.

Hyper-crosslinking: 15 g of polymer was suspended in 100 mL of dichloroethane. After 30 min, 20 mL of stannic chloride was added and the mixture was stirred for 5 h and then left for another 24 h. The polymer was then filtered and washed thoroughly with ethanol.

#### 2.2.2. Preparation of Dendrimer

**Dendrimer-(COOMe)_4_:** In a solution of 0.7 g tetraallyl silane (3.64 mmol, 1 equiv.) in dry DMF (6 mL), a catalytic amount of 2,2-Dimethoxy-2-phenylacetophenone (DMPA) was added. The reaction mixture was degassed by bubbling argon for 5 min. After addition of 2.32 g of methyl thioglycolate (0.0218 mol, 4.4 eq.), the solution was degassed again (1 min). The reaction mixture was then exposed to UV light for 30 min. The solvent and excess methyl thioglycolate were then evaporated under reduced pressure. The crude product was dissolved in diethyl ether and extracted with 1% of NaOH solution (3×), water (2×), and finally brine (1×). The organic layers were collected, dried over MgSO_4_, filtered, and evaporated. Crude product was purified using column chromatography (PE:EtOAc 1:4 → 1:2) and isolated as viscous yellow liquid (1.57 g, 87%).

Data for **1**: NMR (CDCl_3_): ^1^H NMR (400 MHz) δ 0.56–0.61 (m, 8H, SiC*H*_2_), 1.46–1.56 (m, 8H, SiCH_2_C*H*_2_), 2.58 (t, *J* = 7.2 Hz, 8H, SiCH_2_CH_2_C*H*_2_), 3.31 (s, 8H, SC*H*_2_COOMe), 3.63 (s, 12H, *Me*). ^13^C NMR (101 MHz) δ 11.2 (Si*C*H_2_), 23.3 (SiCH_2_*C*H_2_), 32.6 (*C*H_2_COOMe), 35.6 (SiCH_2_CH_2_*C*H_2_), 52.0 (*Me*), 170.7 (*C*O), and ^29^Si NMR (79 MHz) δ 3.66.

HRMS (ESI): *m*/*z* [M + H] ^+^ theoretical for [C_24_H_45_O_8_S_4_Si]^+^:617.1761; found:617.1759.

**Dendrimer:** In a 0.5 g of dendrimer-(COOMe)_4_ (0.81 mmol), ethylenediamine was added under argon in large excess (10 mL). Reaction mixture was then stirred for 12 h (70 °C). Excess ethylenediamine was subsequently evaporated to obtain the product as viscous liquid (0.681 g, 98%).

Data pro **4**: NMR (DMSO-*d_6_*): ^1^H NMR (400 MHz, H-H COSY): 0.55–0.59 (m, 8H, SiC*H*_2_), 1.45–1.53 (m, 8H, SiCH_2_C*H*_2_), 2.55 (t, *J* = 7.5 Hz, 8H, SiCH_2_CH_2_C*H*_2_), 2.57 (t, *J* = 6.2 Hz, 8H, C*H*_2_NH_2_), 3.04 (td, *J* = 6.2, 5.7 Hz, 8H, NHC*H*_2_), 3.08 (s, 8H, C*H*_2_CO), 8.0 (t, *J* = 5.7 Hz, 4H, N*H*). ^13^C {^1^H} NMR (101 MHz, HSQC, HMBC):11.3 (Si*C*H_2_), 23.5 (SiCH_2_*C*H_2_), 34.3 (CO*C*H_2_), 35.6 (SiCH_2_CH_2_*C*H_2_), 41.2 (*C*H_2_NH), and 42.3 (*C*H_2_NH_2_). ^29^Si {^1^H} NMR (79 MHz): δ 3.56.

HRMS (ESI): *m*/*z* [M + H] ^+^ theoretical for C_28_H_60_N_8_O_4_S_4_Si:729.3462, found: 729.3481.

#### 2.2.3. Functionalization of HCLPP

The prepared hyper crosslinked polymer 0.4 g was suspended in 20 mL of acetonitrile. Amine group-containing reagents (ethylenediamine EDA, diethylenetriamine DETA) or dendrimers (see Figure 1b, left) 0.2 g were added dropwise (0.1 mg in 2 mL of acetonitrile). Reaction mixture was then stirred at 50 °C overnight (12 h). After cooling to room temperature, the reaction mixture was centrifuged, washed twice with acetonitrile, and then dried under reduced pressure. Subsequently, the solid was suspended in 5 mL of 10% K_2_CO_3_. After centrifugation, the samples were washed twice with water and dried under reduced pressure.

### 2.3. Characterization

#### 2.3.1. Properties of the HCLPPs

All forms of the prepared hyper-crosslinked materials were characterized using the following methods: Textural characteristics, including the specific surface area, pore volume, and pore diameter distribution, were determined based on nitrogen adsorption-desorption isotherms measured at 77 K using a commercial automated volumetric gas adsorption analyzer ASAP 2050 (Micromeritics, Norcross, GA, USA). Before the measurements, the samples were degassed under deep vacuum at 373 K for 4 h. The morphology and elemental composition were evaluated using a scanning electron microscope (SEM) equipped with an energy-dispersive X-ray (EDX) spectrometer (Quantax, Bruker, Bremen, Germany) in the range of 5–30 kV. Using this technique, the films were analyzed at an accelerating voltage of 15 kV. Thermogravimetric analysis (TGA) of samples was performed using a TG 209 F1 Libra equipment (NETZSCH, Selb, Germany) in the temperature range from 25 °C to 1000 °C in an inert argon atmosphere with a flow rate of 50 mL⋅min^−1^. The samples were heated from ambient temperature to 1000 °C at a heating rate of 4 °C min^−1^, the weight of each sample was approximately 7 mg.

#### 2.3.2. Characterization of Dendrimers

NMR spectra were measured using a Bruker Avance 400, Bruker, Bremen, Germany (^1^H at 400.1 MHz; ^13^C at 100.6 MHz; ^29^Si {^1^H} (INEPT technique) at 79.5 MHz) at 25 °C. ^1^H and ^13^C NMR signals of the prepared compounds were assigned to the corresponding atoms using gHSQC, gCOSY, and gHMBC 2D NMR correlation spectra. ^1^H and ^13^C chemical shifts (δ/ppm) are given relative to residual solvent signals (δ_H_/δ_C_: DMSO-*d6* 2.50/39.52); ^29^Si spectra were referenced to an external standard hexamethyldisilane (−19.87 ppm). HRMS spectra were measured using a MicroTOF-QIII instrument (Bruker Daltonics, Bruker, Bremen, Germany) with an ESI or APCI ionization source in the positive mode.

### 2.4. Gas Adsorption Measurement

**Figure 2:**
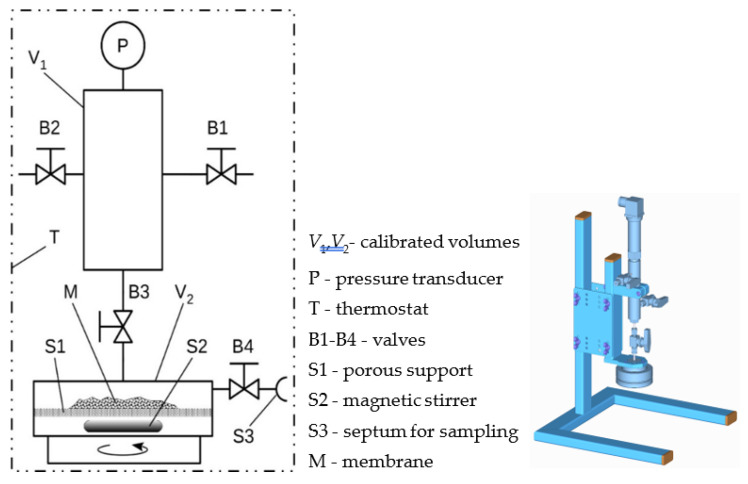
Design and schema of the experimental apparatus for gas adsorption capacity measurement.

The adsorption isotherms of the tested gases were measured using the volumetric method. The adsorption of pure gas in the prepared materials was measured using a simple apparatus shown in Figure 2 constructed in our laboratory.

The thermostated part of the apparatus consisted of two chambers with calibrated volumes, *V*_1_ and *V*_2_, separated by a valve. The pressure in the apparatus was measured using a pressure transducer. A heated box maintains a constant temperature of the entire system during the absorption experiment.

Before the experiment, a weighed amount of the sample was placed in the adsorption chamber, *V*_2_. First, to remove dissolved gases and impurities, the apparatus chambers were evacuated at least 5 h at temperature 80 °C. Subsequently, the system temperature was set to the thermostat at the required experimental value. Chamber *V*_2_ was closed, while volume *V*_1_ was filled with the studied gas at the desired pressure *p*_1_ (from zero to approximately 400 kPa). At the start of the sorption measurement, volumes *V*_1_ and *V*_2_ were connected, and the pressure inside the apparatus was monitored by a computer. The pressure initially dropped immediately to a certain value because of the interconnection of the chambers. Subsequently, the pressure decreased further, albeit more slowly, owing to the adsorption of gas in the sample material. The time required to attain equilibrium was approximately 3 h.

The amount of gas adsorbed in the material was calculated from the balance as the difference between the initial and equilibrium amounts of gas in the system:

(1) $$N_{\left(adsorbed\right)}=N_{\left(gas\;initially\right)}-N_{\left(rest\;gas\;in\;equilibrium\right)}$$

(2) $$N_{\left(adsorbed\right)}=\frac{p_{1}V_{1}-p_{eq}\left(V_{1}+V_{2}-V_{x}\right)}{RT}$$

The ideal gas state Equation (3) is used for the moles in the gas-phase calculation (*N* gas mole number):

(3) $$N=\frac{pV}{RT}$$

The adsorbed amount is related to the material weight *m* to determine the adsorbed phase concentration *q*:

(4) $$q=\frac{N_{\left(adsorbed\right)}}{m}$$

## 3. Results and Discussion

### 3.1. Properties of HCLPPs

The textural properties of these materials are listed in Table 1. The results revealed that the pristine hyper-crosslinked porous polymer had the highest Brunauer-Emmett-Teller surface area (*S_BET_* = 757 m^2^ g^−1^) and its structure was partially microporous and mesoporous.

The reaction of the prepared porous material with polyamine species (amines and amine groups containing dendrimers) decreased the specific surface area and total volume of pores, while in the case of modification by dendrimers, the decrease was smaller than that for amines. The reduction in specific surface area due to amine functionalization was also reported by Lee et al. [32], see Table 6.

Similarly, the total and micropore volumes decreased significantly after amine modification. However, the modification by dendrimers reduced the total and micropore volumes only slightly, which indicated difficulties in filling the micropores with voluminous dendrimer molecules affecting only the region of mesopores and not the micropores.

**Figure 3:**
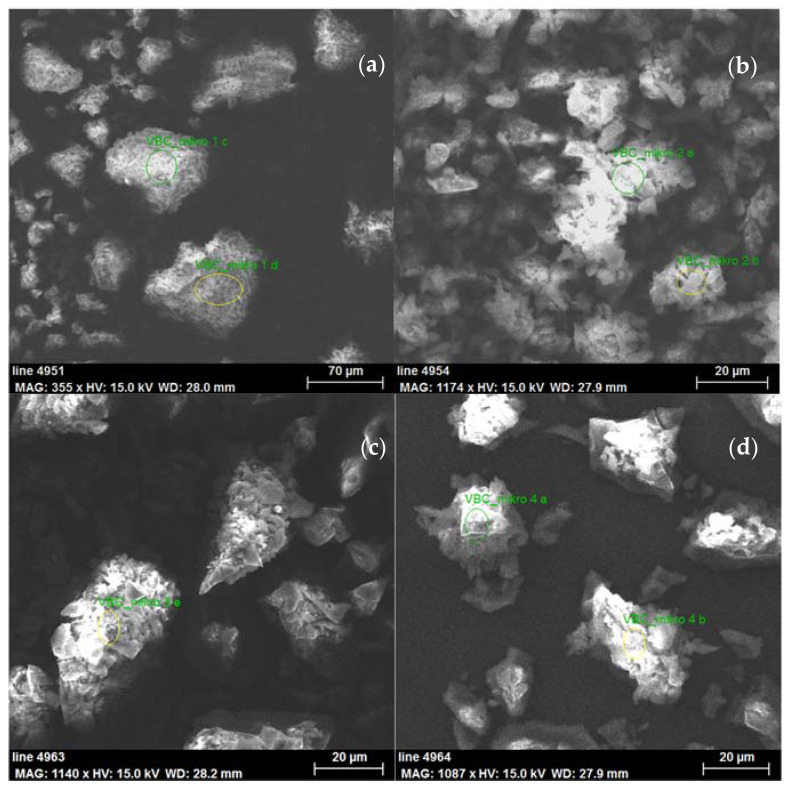
SEM images of synthesized polystyrene based HCLPPs (**a**) pristine HCLPP, (**b**) HCLPP-EDA, (**c**) HCLPP-DETA, and (**d**) HCLPP-DENDRIMER, respectively.

To investigate the morphology and elemental composition of the prepared HCLPP, a SEM equipped with an EDX spectroscopy detector was used. From the photographs in Figure 3, the morphologies of all prepared polymers were similar, and the resulting materials had different particle sizes of irregular shape with no observable changes after functionalization by agents containing amino groups.

Elemental analyses of the prepared polymers, performed using EDX, are given in Table 2. After functionalization, the nitrogen content in the materials considerably increased. The modification by amines increased the nitrogen content to more than 9%, while with the dendrimer, the value of approximately 5% was achieved. Simultaneously, the modification lowered the chlorine content, indicating a reaction with the chloride units. The results show a more effective reaction and exchange of chloride groups for amine groups and a significantly higher density of amine groups on the surface of the porous material for amine functionalization compared to that of modification with a dendrimer. These findings were in agreement with the morphological data shown in Table 1.

Elemental analysis of the dendrimer-modified sample also revealed the presence of sulfur and silicon in the modifying agent. A small amount of silicon was also detected in other porous polymers, which was probably caused by contamination during the grinding of the porous samples in the ceramic bowl before the analyses.

**Figure 4:**
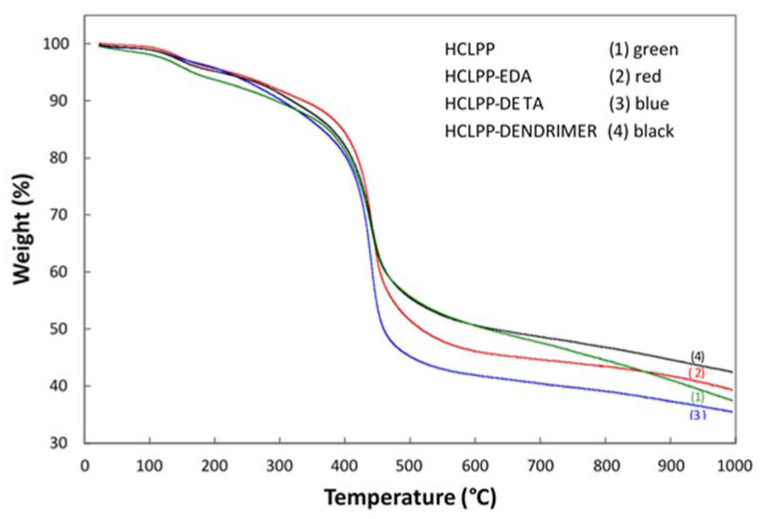
TGA curves of synthesized polystyrene based HCLPPs.

Thermal stability of polymers was evaluated by TGA in the temperature range of 25 to 1000 °C under argon atmosphere with heating rate of 4 °C min^−1^. The TGA results are shown in Figure 4. The profiles of all the samples were very similar. A weight loss of approximately 6% at a lower temperature up to 250 °C was observed, probably caused by the evaporation of water, residual solvent, and trapped gases in the porous structure of the material. Significant weight loss followed as the temperature increased to 445 °C, corresponding to the decomposition of polystyrene-based polymers [28,40].

Compared to waste expanded PS undergoing complete weight loss in the range of 400–440 °C [27,40], the prepared porous polymers were more thermally stable, having a total weight loss at 1000 °C of only approximately 58% to 65%. This high thermal stability is because of the rigid cross-linked porous structure formed during the Friedel-Crafts reaction [27,41].

### 3.2. Gas Adsorption and Selectivity

The adsorption isotherms of gases (CO_2_, CH_4_, N_2_, H_2_, and O_2_) were measured using a simple volumetric sorption apparatus designed in our laboratory (the scheme is given in Figure 2). All sorption measurements were performed at a constant, actively controlled temperature of 25 °C.

Table 3 presents a comparison of the adsorption capacities of the unmodified and modified polymers for pure gases, determined from the measured isotherms at a pressure of 100 kPa.

The results demonstrated that polymer modification by amines led to a decrease in the amount of adsorbed gas, with the exception of oxygen. This could be attributed to the reduced surface area of the amine-functionalized forms of the polymer. However, the decrease in the adsorption capacity was not as significant as the decrease in the specific surface area or pore volume of the modified materials. Lee et al. [32] reported similar trends in CO_2_ and N_2_ capacities at 25 °C for the porous polymer network synthesized by solvothermal polymerization of DVB and VBC, see Table 6.

In contrast, in the case of the material modified with a dendrimer containing amino groups, an increase in the adsorption performance of CO_2_ and oxygen was observed, despite the fact that, similar to other modified forms of the polymer, the specific surface area also decreased, but to a lesser extent.

However, if the adsorption capacities of the materials were normalized to the specific surface area, the efficiency of CO_2_ capture was significantly improved, thus confirming the positive effect of functionalization (Table 4).

While the gas-uptake ability of the porous material decreased after functionalization, the selectivity increased in all cases. In particular, the CO_2_/N_2_ and O_2_/N_2_ separation efficiencies of the HCLPPs after amination were improved. Table 5 shows the selectivity values for different gas pairs obtained from the adsorption isotherms of pure gases at 100 kPa.

**Figure 5:**
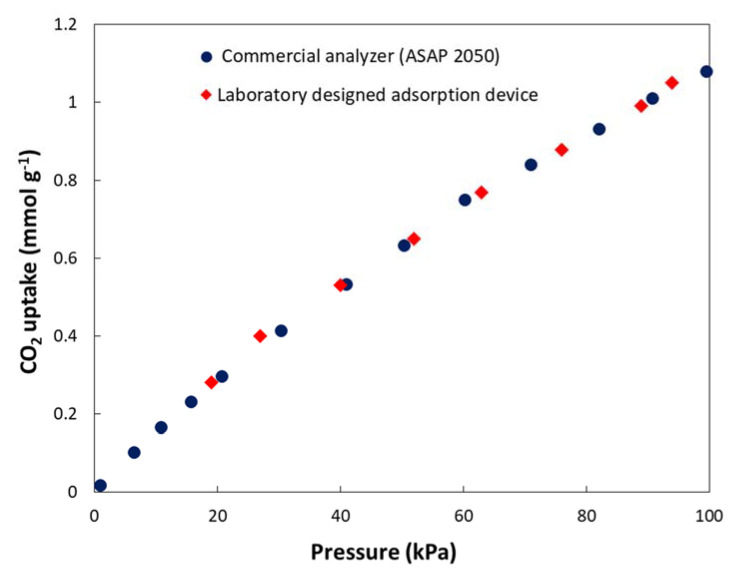
CO_2_ adsorption on the unmodified HCLPP measured at 25 °C using the laboratory constructed device and commercial apparatus ASAP 2050.

To verify the precision of the measurements using the simple apparatus constructed in our laboratory, we also performed control measurements of CO_2_ adsorption using the automated commercial apparatus ASAP 2050 (Micromeritics, Norcross, GA, USA). As shown in Figure 5, the agreement of the results was excellent; therefore, the apparatus provided the correct results.

The obtained CO_2_ adsorption data of the prepared materials were compared with other hyper-crosslinked porous polystyrene-based polymers studied for CO_2_ capture application reported in literature (Table 6), for examples HCLPs prepared via the Friedel-Craft reaction between waste expanded polystyrene and 1,2-dichloroethane as both the solvent and the crosslinking agent [27], similar polymers produced using four cross-linkers in order to enhance the porosity of the polymeric adsorbent [28], or amine-functionalized porous polymer network prepared by conventional radical polymerization. In all cases, the material prepared in our study showed a comparable or slightly lower CO_2_ adsorption capacity, but in most cases the higher selectivity.

**Table 6:** CO_2_ capture and selectivity adsorption performance of some styrene-based polymers.

Sample	*S_BET_*, (m^2^ g^−1^)	CO_2_, (μmol g^−1^) 1 bar, 273 K, 298 K		CO_2_/N_2_ Selectivity	Ref.
HCP-3A	179	932		9	
HCP-6A	299	1604		22	
HCP-12A	406	1987	273 K	23	[27]
HCP-24A	548	1715		20	
HCP-12B	488	1771		20	
HCP-12C	573	1846		15	
HCP-0	425	1201		17	
HCP-A	777	1473		38	
HCP-B	768	1178	298 K	25	[28]
HCP-C	611	1258		15	
HCP-D	673	1359		15	
P(DVB-VBC)-31	744	390		20	
P(DVB-VBC)-31-EDA	389	1150		152	
P(DVB-VBC)-31-DETA	376	1440	298 K	223	[32]
P(DVB-VBC)-11	134	180		-	
P(DVB-VBC)-11-EDA	91	1150		-	
P(DVB-VBC)-11-DETA	48	750		-	
HCLPP	757	1010		28	
HCLPP-EDA	283	910	298 K	81	This work
HCLPP-DETA	277	820		71	
HCLPP-DENDRIMER	616	1100		29	

## 4. Conclusions

In summary, we presented a method of preparation of a new porous polymeric adsorbent by the suspension polymerization of divinylbenzene and 4-vinylbenzyl chloride, followed by modification with amines and branched dendrimers containing amino groups in order to enhance its CO_2_ affinity. Synthesized hyper-crosslinked porous material showed a high apparent surface area and selective CO_2_ adsorption over CH_4_, N_2_ and H_2_, which significantly increased after amines functionalization. Therefore, produced porous polymers represent promising organic porous materials for CO_2_ uptake from gas mixtures. The results indicated that the reaction of the prepared polymer with the dendrimer was not as effective as its reaction with amines due to the partially microporous structure being too tight for the possible interaction with the branched dendrimer molecules. Based on this finding, we will focus in the future on the development of polymers with mesoporous structure that may be more suitable for dendrimer loading. The prepared porous polymer structure and its amine-functionalized forms are considered for testing as fillers into mixed-matrix membranes with improved CO_2_/CH_4_ and CO_2_/N_2_ separation performance as well.

## Figures and Tables

**Table 1:** Texture properties of pure and functionalized porous polymers HCLPPs.

Sample	*S_BET_*^a^ (m^2^ g^−1^)	*S_meso_*^b^ (m^2^ g^−1^)	*V_tot_*^c^ (mm^3^_liq_ g^−1^)	*V_micro_*^d^ (mm^3^_liq_ g^−1^)
HCLPP	757	310	408	226
HCLPP-EDA	283	93	157	97
HCLPP-DETA	277	132	159	76
HCLPP-DENDRIMER	616	194	341	218

^a^ *S_BET_* the specific surface area calculated by the BET method from the N_2_ adsorption isotherm at T = 77 K; ^b^ *S_meso_* is the specific surface area of mesopores (t-plot method using the standard isotherm according to Harkins and Jura [39]); ^c^ *V_tot_* the specific total volume of pores determined from the N_2_ adsorption isotherm at *p*/*p*^0^ = 0.99; ^d^ *V_micro_* the specific volume of micropores (t-plot method determined by the t-plot method using the standard isotherm according to Harkins and Jura).

**Table 2:** EDX analysis of pure and functionalized porous polymers HCLPPs.

Sample	C (%)	Cl (%)	N (%)	S (%)	Si (%)
HCLPP	97.79	0.42	1.74	-	0.05
HCLPP-EDA	90.62	0.08	9.21	-	0.10
HCLPP-DETA	90.55	0.05	9.32	-	0.07
HCLPP-DENDRIMER	99.93	0.30	5.37	0.20	0.21

**Table 3:** Gas adsorption capacity of pure and functionalized porous HCLPP at 100 kPa and 25 °C.

Sample	CO_2_ (μmol g^−1^)	CH_4_ (μmol g^−1^)	N_2_ (μmol g^−1^)	H_2_ (μmol g^−1^)	O_2_ (μmol g^−1^)
HCLPP	1010	310	90	21	12
HCLPP-EDA	910	130	33	15	47
HCLPP-DETA	820	130	29	12	42
HCLPP-DENDRIMER	1100	240	63	17	89

**Table 4:** Gas adsorption capacity of pure and functionalized porous HCLPP based on VBC *.

Sample	CO_2_ (μmol m^2^)	CH_4_ (μmol m^2^)	N_2_ (μmol m^2^)	H_2_ (μmol m^2^)	O_2_ (μmol m^2^)
HCLPP	1.33	0.41	0.12	0.03	0.02
HCLPP-EDA	3.22	0.46	0.12	0.05	0.17
HCLPP-DETA	2.96	0.47	0.10	0.04	0.15
HCLPP-DENDRIMER	1.79	0.39	0.10	0.03	0.14

* at temperature 25 °C and pressure 100 kPa.

**Table 5:** Selectivity of pure and functionalized porous polymers HCLPP based on VBC *.

Sample	CO_2_/CH_4_ (-)	CO_2_/N_2_ (-)	CO_2_/H_2_ (-)	O_2_/N_2_ (-)	CH_4_/N_2_ (-)
HCLPP	5.8	27.6	74.2	0.1	4.7
HCLPP-EDA	16.0	81.0	188.9	1.4	5.1
HCLPP-DETA	12.8	71.4	172.6	1.5	5.6
HCLPP-DENDRIMER	6.2	28.9	109.3	1.4	4.7

* at temperature 25 °C and pressure 100 kPa, calculated by IAST.

## Data Availability

Not applicable.
